# Case of Colloboma Iridis

**Published:** 1850-09

**Authors:** A. Garwood

**Affiliations:** Cassoppolis, Mich.


### ARTICLE VII.

*Case of Colloboma Iridis.*—By A. Garwood, M. D., of Cassoppolis, Mich.

The following case lately came under my observation, which was peculiarly novel and interesting to me, and I thought it might also be to oculists and the profession generally.

It was a case of congenital Coloboma Iridis, in a beautiful little girl, 10 years of age. The fissure extended from the pupil to the inferior ciliary margin of the iris. It gave to the eye a peculiar, staring appearance. She was troubled with Myopeia, but could see as well as usual for those that are short-sighted. I supposed from the largeness of the pupils that her vison would be better after night than persons generally, but her mother stated that she never observed that it was. The iris vibrates upon every motion of the aqueous humor, and it appears very much attenuated.

